# Knowledge, Attitude, and Practices of Labor Analgesia Amongst Obstetric Residents: A Cross-Sectional Survey

**DOI:** 10.7759/cureus.62326

**Published:** 2024-06-13

**Authors:** Swati Vijapurkar, Gade Sandeep, Jitendra V Kalbande, Sandra Merin Thomas, Subrata K Singha

**Affiliations:** 1 Anesthesiology, All India Institute of Medical Sciences, Raipur, Raipur, IND; 2 Cardiac Anesthesia, All India Institute of Medical Sciences, Raipur, Raipur, IND; 3 Anesthesiology and Critical Care, All India Institute of Medical Sciences, Raipur, Raipur, IND

**Keywords:** labor, labor analgesia, anesthesiologist, regional analgesia, opioids

## Abstract

Background: Labor analgesia plays a crucial role in ensuring a comfortable and positive birthing experience. It provides physiological benefits to both the mother and the child. Knowledge, awareness, and communication between the anesthesiologist and the obstetrician are essential for the safe conduct of labor analgesia. We conducted this cross-sectional online survey amongst obstetric residents to assess their knowledge, attitude, and practices of labor analgesia.

Methods: A structured questionnaire consisting of 19 questions was circulated amongst obstetric residents of various hospitals via electronic mode of communication. The responses were analyzed using statistical methods.

Results: Among the obstetric residents that we surveyed, 75.7% of them only sometimes employed labor analgesia for their patients. The most commonly employed methods of pain relief are opioids and non-steroidal anti-inflammatory drugs (NSAID). Most of them feel that pain-free labor is necessary because it makes the whole labor process a pleasurable one. Labor analgesia was mostly advocated at patients' request and demand. The barrier to using labor analgesia was most commonly found to be the non-availability of labor analgesia services.

Conclusion: Despite the increasing awareness of labor analgesia there still lies a gap between the attitude toward it and the practice of it. Further education to rectify the misconceptions and barriers needs to be taken for providing beneficial services to pregnant females.

## Introduction

Labor pain is a complex sensation caused by uterine contractions and the stretching of the birth canal. It has both physiological and pathological mechanisms. Labor pains are typically rated as one of the worst pains a woman can ever experience in her lifetime [1].

Nulliparous women experience greater pain as compared to multiparous women. In the first stage, the labor pain is mediated by T10 to L1 spinal segments, whereas in the second stage, it is carried by T12 to L1, and S2 to S4 spinal segments [2].

Labor analgesia, the relief of this pain through various medical techniques, plays a crucial role in improving the birthing experience for mothers by reducing discomfort, stress, and anxiety. Labor analgesia promotes maternal well-being, facilitates the progress of labor, and enhances the overall childbirth experience for both the mother and baby.

Methods of pain relief in labor include pharmacological and non-pharmacological techniques. The non-pharmacological techniques include touch and massage, acupuncture, water baths, intradermal sterile water injections, and transcutaneous electrical nerve stimulation (TENS). The pharmacological techniques include parenteral narcotics, inhalational methods, and neuraxial techniques [3].

The objective of this survey was to assess the knowledge, attitude, and practices of labor analgesia amongst obstetric residents.

## Materials and methods

A cross-sectional survey was conducted among obstetric residents belonging to various institutes. The survey was conducted via Google Forms that was circulated online. The responses of those obstetricians willing to fill out the forms were included.

A structured questionnaire having four components that included sociodemographic parameters, knowledge, attitude, and practice-related questions was developed. The structure of the questionnaire was aimed at assessing the knowledge and affective component of the participants based on the three domains of learning which include cognitive knowledge, attitude, and psychomotor skills [4].

The questionnaire contained 19 questions in English with all the questions requiring a mandatory response. The questionnaire was designed by the authors based on a previous study by Melesse et al. [5]. The questionnaire was reviewed by five anesthesiologists who have knowledge about labor analgesia.

The survey was conducted after obtaining informed consent from the participants. Only one response was allowed from each participant and the confidentiality of the records was maintained.

No prior sample size calculation was determined. This survey was conducted over two weeks and the number of responses received during this period was included in the study.

The inclusion criteria include obstetric residents willing to participate and submit their responses, residents belonging to either government or private hospitals, and both junior residents and senior residents.

The exclusion criteria include obstetric residents not willing to participate, residents in administrative roles rather than clinical residency roles, and residents unable to comprehend the language of the questionnaire which was in English.

The operational definitions are as follows: often is defined as more frequently applied but not always, while sometimes is defined as less frequently applied.

Statistical analysis

Data from the Google Sheets was analyzed using IBM SPSS Statistics for Windows, Version 23 (Released 2015; IBM Corp., Armonk, New York, United States). The study was analyzed using frequencies and percentages along with a chi-square test. A value of p <0.05 was considered to be significant.

## Results

Out of the 145 forms distributed, 74 responses were recorded with a response rate of 51.03%. Among the obstetric residents that we surveyed 13.5% (n=10) of them were first-year residents, 32.4% (n=24) were second-year residents, 40.5% (n=30) were third-year residents and 13.5% (n=10) were senior residents.

Eighty-nine (89.2%; n=66) percent of them belonged to government institutions while the remaining (10.8%; n=8) belonged to private institutions.

In the questions asked to assess their knowledge, only 56.8% (n=42) of them were familiar with the World Health Organization (WHO) analgesic step ladder. Forty percent (40.5%; n=32) of them employed pain scores for the classification of pain.

Routinely used methods of pain relief for the management of labor pains were most commonly opioids (29.7%; n=22) followed by non-steroidal anti-inflammatory drugs (NSAID) (27%; n=20). Non-pharmacological techniques of labor analgesia were used by 8% (n=6) of them while 5% (n=4) of them used regional anesthetic techniques. The remaining residents used a combination of these techniques.

**Figure 1:**
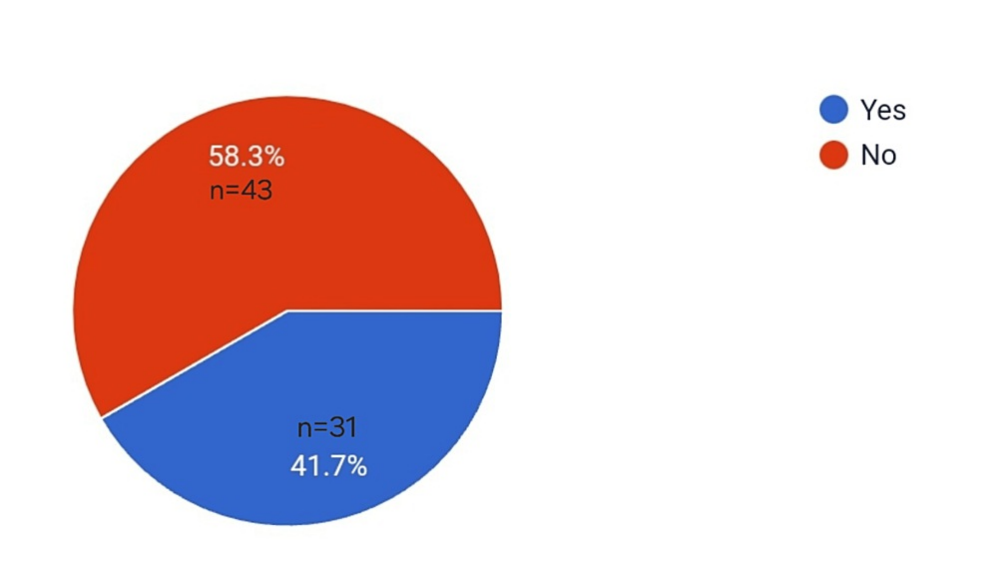
Pie chart depicting the percentage of routine counseling regarding pain-free labor given for pregnant females coming to regular check-ups

The following pie chart depicts the percentage of routine counseling regarding pain-free labor given to pregnant females coming for regular check-ups (Figure 1).

Pain-free labor was considered necessary since the majority of them felt that it makes the whole labor process more pleasurable and makes the patient more cooperative (73%; n=54). The least of them felt that it improves placental perfusion and decreases fetal acidemia and hypoxia (18.9%; n=14). Since multiple answers could be given to this question, the remaining residents chose a combination of the answers.

Labor analgesia was most commonly advocated at the request and demand of pregnant females (75.5%: n=56). Due to certain misconceptions, eight (10.8%; n=8) of the residents chose to advocate labor analgesia in patients with deranged coagulation profiles.

**Figure 2:**
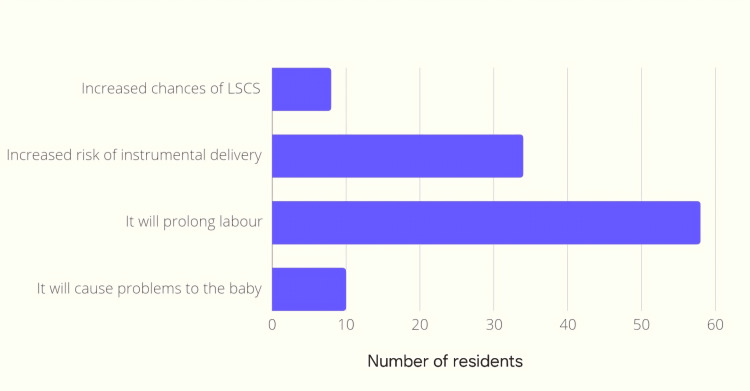
Bar graph depicting the concerns of using labor analgesia LSCS: lower segment cesarean section

In the next question regarding concern about using labor analgesia, multiple answers were allowed to be chosen. Prolongation of labor was the most common concern for using labor analgesia (80.6%) (Figure 2).

Amongst the 74 residents, 52 (70.3%) of them "sometimes" approach the anesthesiologist for labor analgesia while 16% (n=12) of them never approach the anesthesiologist. Eight percent (8.1%; n=6) of them always approach the anesthesiologist.

Labor analgesia is deployed on maternal demand (89.2%; n=66) rather than routinely given in their institute.

**Figure 3:**
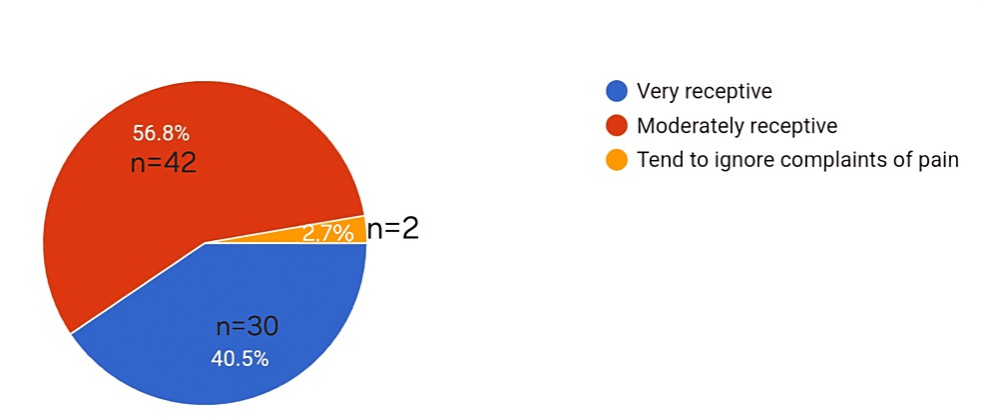
Pie chart depicting the percentage of residents receptive toward the patients' pain

The following pie chart depicts the percentage of residents receptive to the patients' pain (Figure 3).

**Figure 4:**
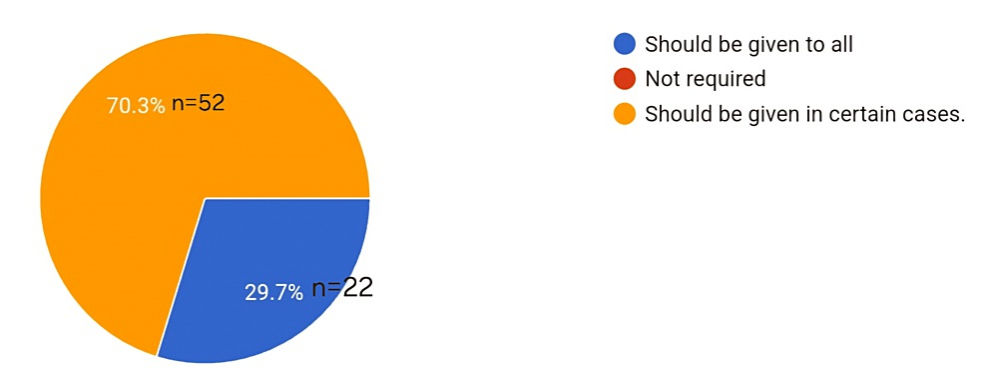
Pie chart depicting the requirement of labor analgesia

In Figure 4, the pie chart shows the opinion of the residents regarding the requirement for labor analgesia (Figure 4).

Ninety-seven (97.3%; n=72) percent of the residents are willing to learn about labor analgesia while 2.7% (n=2) of them are unwilling to learn about labor analgesia.

In questions related to the practice of labor analgesia, 8.1% (n=6) of the residents have never employed labor analgesia to their patients while 75.7% (n=56) of them have only "sometimes" employed labor analgesia.

**Figure 5:**
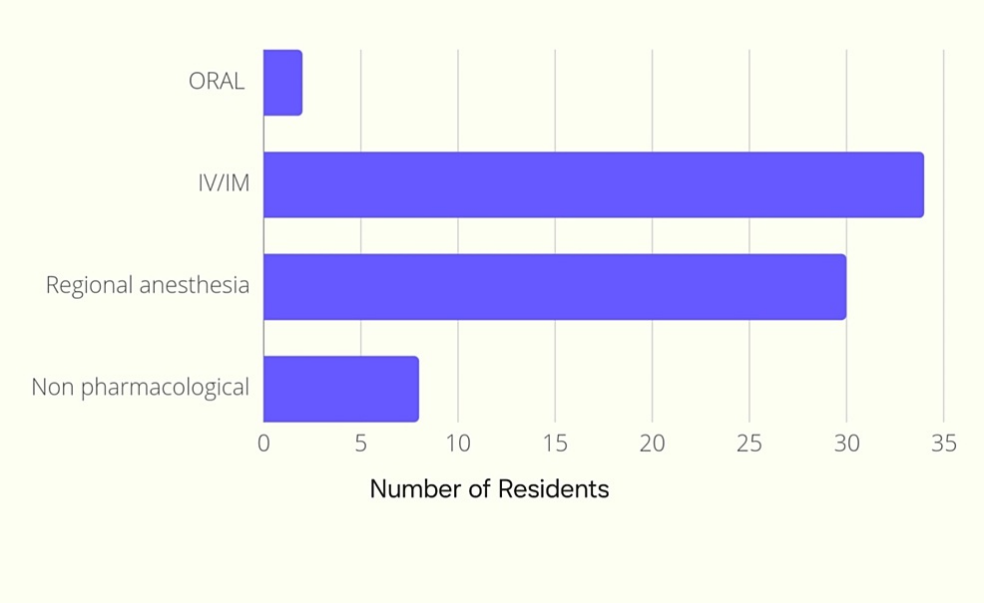
Bar graph depicting the type of labor analgesia preferred IV: intravenous; IM: intramuscular

Intravenous and intramuscular types of labor analgesia were most commonly preferred (45.9%; n=34) followed by regional anesthetic techniques (40.5%; n=30) (Figure 5).

Preference for the above technique was due to the ease of administration while the non-availability of labor analgesia services in most of the centers was a hindrance to the use of regional techniques.

The complications encountered were most commonly decreased/no pushing efforts by the mother (40.5%; n=30). Instrumental delivery/cesarean section was encountered in 29.7% (n=22) of the cases. Around 18.9% (n=14) of them encountered inadequate analgesia, and 5.4% (n=4) encountered fetal complications. The remaining 5.4% (n=4) didn't encounter any complications.

The barriers to using labor analgesia were most commonly due to the unavailability of a labor analgesia team (37.8%; n=28). The chi-square test was applied to determine the statistical significance of this response and it was found to be statistically significant (p= 0.000) as compared with the other barriers to using labor analgesia (Table 1).

**Table 1:** Table showing barriers to using labor analgesia and p-value

Barriers to using labor analgesia	Frequency	Percent	χ^2^	p-value
Fear of complications	20	27.0%	27.89	0.000
Financial constraints	2	2.7%		
Lack of awareness	16	21.6%		
Non-availability of labor analgesia/anesthesia team	28	37.8%		
Patients’ refusal and preference for cultural norms	8	10.8%		
Total	74	100.0%		

## Discussion

Labor pain is caused by the rhythmic contractions of the uterus. It is often described as intense and can vary in duration and intensity among different women. Labor pain evokes a metabolic, systemic, and neuro-humoral stress response causing enormous physiological effects. Respiratory effects like hyperventilation leading to alkalosis, and increased oxygen consumption, cardiovascular effects such as raised blood pressure, cardiac output and heart rate, delayed gastric emptying time, and decreased uterine blood flow [6]. Effective pain management strategies can help mitigate these adverse effects and promote a more positive birthing experience.

Labor analgesia refers to the administration of pain relief during childbirth to alleviate the discomfort experienced by the mother. Various methods of pain relief during labor include non-pharmacological techniques and pharmacological techniques [7]. Many neuraxial techniques have been introduced in recent years, with the use of low-dose opioids that have improved the quality of analgesia [8].

In a study by Narayanappa et al. [9], the highest rate of labor analgesia practice was in the private setup and least by government hospitals. In our study, most of the residents belonged to government institutions and reported the use of labor analgesia less frequently.

A little more than half of the residents we surveyed knew the WHO analgesia ladder (56%) but most of them didn't employ any pain scores. Similar findings in a study by Melesse et al. [5], showed that 88.4% know about the WHO pain ladder, but a lesser percentage use it to treat pain, showing that there seems to be a gap between the knowledge and application of it. This could also be the reason why most of the residents were only moderately receptive to patients’ pain and 2.7% of them tend to ignore the complaints of pain.

In our study, the most commonly used methods were opioids, NSAIDs, and preferred IV/IM techniques similar to the study by Bishaw et al. [10], and regional anesthetic techniques were less commonly used. In a study by Ali et al. [11], to assess the knowledge, attitude, and practices of labor analgesia amongst healthcare workers and patients, systemic opioids with adjuvants were the most common methods of pain relief used in labor. This could be influenced by their lack of awareness and fear of complications like decreased/no pushing efforts by the mother, instrumental delivery/cesarean section, and fetal complications. This could also be the reason why the residents only "sometimes" employed labor analgesia for their patients. Routine counseling to all pregnant females was not given, which could lead to a lack of awareness among the patients similar to the study by Sharma et al. [12], on pregnant females where only 16% had awareness of labor analgesia.

In the question of why pain-free labor was considered necessary, the least of them felt that it improves placental perfusion, decreases fetal acidemia and hypoxia, or reduces the sympathetic response showing poor knowledge about the physiological effects of labor pains and the benefits of attenuating it. Labor analgesia was most commonly advocated based on patients’ request and demand while other indications like pregnancy-induced hypertension and sickle cell disease were less chosen. Awareness regarding the indications and benefits of labor analgesia in other conditions is lacking and knowledge about this must be enhanced.

Prolongation of labor was the most common concern while using labor analgesia. In the study by Melesse et al. [5], the majority of their participants felt that their concern was the effect on the baby's breathing followed by a prolongation of labor, while in our study neonatal complications were chosen by a lesser number of residents. Similar results were found in a study conducted by Khan et al. [13], to assess the perception, attitude, and practice of labor analgesia among obstetric care providers in Eastern Uttar Pradesh, 26% of the participants cited prolongation of the labor process as the most common problem perceived with the use of the epidural technique. The majority of the residents only "sometimes" approach the anesthesiologist, which could also be a barrier to poor employment of labor analgesia, and this emphasizes the importance of communication between the obstetrician and the anesthesiologist.

The response of 97.3% of residents willing to learn about labor analgesia is a positive sign for improving labor analgesia services and making it available for a larger pregnant population. The barriers to using labor analgesia were most commonly due to the unavailability of a labor analgesia team and this was found to be statistically significant (p= 0.000). This was similar to a study by Ponnusamy et al. [14] where obstetricians felt the non-availability of the anesthesiologist was the main barrier to the use of labor analgesia. Various studies among pregnant women to assess knowledge, attitude, and use of labor analgesia found that the majority of the women wanted to experience natural childbirth as the reason why they did not want labor analgesia [15,16].

The limitations of this study are the lesser number of total responses received over two weeks. The inclusion of more obstetricians in the study would have given a better idea about the awareness and practices of labor analgesia. The majority of the responses received were from government institutions and fewer responses were from private institutions where labor analgesia is more commonly practiced. More participation from residents belonging to private institutes would have varied the results.

## Conclusions

Despite the increasing popularity of labor analgesia and its proven benefits, there are still certain misconceptions and a lack of knowledge regarding the physiological effects of labor pains and the benefits of labor analgesia. Communication between the obstetrician and the anesthesiologist must be improved, and further education regarding the different methods, and benefits of labor analgesia must be provided.

The unavailability of a labor analgesia team seems to be the major barrier and measures to set up labor analgesia clinics must be considered. Misconceptions and fear of complications must be overcome by education to extend these services for the benefit of every pregnant female.

## Appendices

**Table 2:** Questionnaire for the survey WHO: World Health Organization; NSAID: non-steroidal anti-inflammatory drugs; LSCS: lower segment cesarean section; IV: intravenous; IM: intramuscular The questionnaire was designed by the authors based on a previous study by Melesse et al. [4]. The questionnaire was publicly available to all.

Sl No	Question	Response
SOCIO DEMOGRAPHIC FACTORS		
1.	Year of residency?	● First year ● Second year ● Third year ● Senior resident
2.	Type of Institution in which the residency is being pursued	● Government ● Private
KNOWLEDGE		
3.	Do you have knowledge regarding WHO analgesia ladder?	● Yes ● No
4.	Do you employ any pain scores for classification of pain?	● Yes ● No
5.	What are the methods of pain relief routinely used for Management of labour pains?	● NSAIDs ● Opioids ● Non-pharmacological ● Regional anesthesia
6.	Is counselling regarding pain free labour routinely given for pregnant females coming for regular checkups?	● Yes ● No
7.	Why do you think pain free labour is necessary?	● Decreases the sympathetic response ● Decreases anxiety and fear ● Makes the patient more cooperative ● Decreases maternal demand for LSCS ● Improves placental perfusion and decreases fetal acidemia and hypoxia ● Makes the whole labour process more pleasurable
8.	In which all patients would you advocate labour analgesia?	● Sickle cell disease ● Pregnancy induced hypertension ● On patient's request and demand ● Deranged coagulation profile
9.	What are your concerns of using labour analgesia?	● Increased chances of LSCS ● Increased risk of instrumental delivery ● It will prolong labour ● It will cause problems to the baby
ATTITUDE		
10.	How often do you approach anaesthesiologist for the purpose of labour analgesia?	● Never ● Sometimes ● Often ● Always
11.	How is labour analgesia deployed in your institute?	● On patients demand ● Routinely given
12.	How receptive are you towards the patient's pain?	● Very receptive ● Moderately receptive ● Tend to ignore complaints of pain
13.	In your opinion, is labour analgesia required?	● Should be given to all ● Not required ● Should be given in certain cases
14.	Are you willing to learn about labour analgesia?	● Yes ● No
PRACTICES		
15.	How often do you employ labour analgesia for your patient?	● Never ● Sometimes ● Often given
16.	Which type of labour analgesia do you prefer?	● Oral ● IV/IM ● Regional anesthesia ● Non-pharmacological
17.	Why was the above method preferred over the others?	
18.	What were the complications encountered?	● Inadequate analgesia ● Instrumental delivery/LSCS ● No pushing efforts by the mother ● Fetal complications ● Other:
19.	What are the barriers for using labour analgesia?	● Fear of complications ● Lack of awareness ● Patients' refusal and preference to cultural norms ● Non availability of labour analgesia team ● Financial constraints
